# Physiological and Sleep Profiles in Insomnia, OSA and COMISA: A Comparative Study in Portugal and Spain

**DOI:** 10.1111/jsr.70367

**Published:** 2026-06-05

**Authors:** Margarida Carvalho, Eva Almeida, Pedro Amorim, Marta Drummond, Adrián Martín‐Montero, Gonzalo C. Gutiérrez‐Tobal, Tomás Ruiz‐Albi, Julio De Frutos, Pedro Pereira Rodrigues, Daniela Ferreira‐Santos

**Affiliations:** ^1^ Faculty of Medicine, University of Porto Porto Portugal; ^2^ Sleep and Non‐Invasive Ventilation Unit São João University Hospital Porto Portugal; ^3^ School of Health—P.Porto Porto Portugal; ^4^ Centro de Investigación Biomédica en Red en Bioingeniería, Biomateriales y Nanomedicina, CIBER‐BBN Madrid Spain; ^5^ Biomedical Engineering Group, University of Valladolid Valladolid Spain; ^6^ Biomedical Engineering Group, Tecnología Electrónica, Ingeniería de Sistemas y Automática (TEISA) Department University of Cantabria Santander Spain; ^7^ Pneumology Department Río Hortega University Hospital Valladolid Spain; ^8^ INESC TEC Porto Portugal

**Keywords:** autonomic nervous system, COMISA, heart rate variability, insomnia, obstructive sleep apnea, polysomnography

## Abstract

Insomnia and obstructive sleep apnea (OSA) are common sleep disorders that often co‐occur as comorbid insomnia and sleep apnea (COMISA), worsening health outcomes. This study characterized and compared the physiological and sleep profiles of insomnia, OSA and COMISA in population‐based datasets from Portugal (*n* = 147) and Spain (*n* = 264), excluding patients with cardiovascular disease. Classification followed clinical guidelines, and analyses included polysomnography and heart rate variability (HRV) data. Conventional HRV metrics in the time and frequency domains, along with condition‐specific frequency bands (BWMS, BWOSA, BWCOMISA, BWRes) were compared alongside demographic and clinical variables. The Portuguese and Spanish datasets differed significantly in gender distribution, daytime sleepiness, sleep efficiency and apnea‐hypopnea index (AHI), with Spanish patients showing a higher severity of AHI and a predominantly male composition. COMISA patients were older, had higher BMI, and showed a hypopnea‐dominant respiratory profile in both datasets. Classical HRV indices did not differ significantly between groups, although trends suggested decreased parasympathetic activity in OSA. Condition‐specific bands were largely non‐discriminative in Portugal, while BWOSA emerged as a significant marker of respiratory burden in Spain. When the populations were analysed jointly, age and obesity remained significant risk factors and COMISA maintained its hypopnea‐dominant profile. The combined dataset revealed significant differences in several HRV features. OSA and COMISA patients exhibited a higher cardiovascular burden and autonomic imbalance (lower HF, higher LF/HF and LFn), while condition‐specific bands all showed discriminatory power across groups. BWOSA may represent a useful spectral biomarker for OSA, although population and methodological factors strongly influence discriminative power.

## 1 Introduction

Insomnia and obstructive sleep apnea (OSA) are among the most prevalent and burdensome sleep disorders, frequently co‐occurring in the same individual, a condition known as comorbid insomnia and sleep apnea (COMISA) (Cruz 2024). The co‐occurrence of these disorders was first described by Guilleminault et al. (1973). The term COMISA itself was later coined by Sweetman et al. (2017), and has since been used to describe the simultaneous presence of both disorders, which independently contribute to sleep disruption, reduced quality of life and increased health risks. Diagnosis of COMISA presents a clinical challenge due to overlapping symptoms, such as difficulty falling asleep, frequent nocturnal awakenings, non‐restorative sleep and daytime fatigue. These common features often complicate the identification and treatment of each disorder when both are present (Wulterkens et al. 2023).

COMISA is a highly prevalent disorder, with approximately 39%–58% of individuals diagnosed with OSA also reporting symptoms consistent with insomnia (Sweetman et al. 2017). Individuals tend to experience greater overall impairment than those with either condition alone. This includes poorer subjective and objective sleep quality, more severe daytime dysfunction, heightened stress, reduced productivity, and diminished physical and mental health (Krakow et al. 2001; Watson et al. 2015; Martín‐Montero et al. 2025). Furthermore, COMISA has been associated with increased cardiometabolic risk and elevated mortality rates (Guilleminault et al. 1973).

In addition to the substantial burden, treating COMISA is particularly challenging because interactions between the two conditions may reduce the effectiveness of treatments that work well when each condition occurs alone. Insomnia symptoms often decrease patients' acceptance and long‐term adherence to continuous positive airway pressure therapy. At the same time, uncertainty remains about the optimal sequencing or combination of insomnia and sleep apnea treatments (Sweetman et al. 2017).

HRV is a widely used, non‐invasive marker of autonomic nervous system (ANS) function. HRV reflects the dynamic interplay between the sympathetic nervous system (SNS), which promotes arousal and energy mobilization, and the parasympathetic nervous system (PNS), which facilitates rest and recovery (Rajendra Acharya et al. 2006). In OSA, repeated apneic events cause intermittent hypoxia and sleep fragmentation, leading to sustained increases in SNS activity during sleep, resulting in a reduced HRV and heightened cardiovascular risk (Stein and Pu 2012; Gula et al. 2003; Ha and Kim 2022; Dissanayake et al. 2021). In insomnia, elevated SNS activity is similarly observed, driven by hyperarousal and chronic sleep deprivation (Spiegelhalder et al. 2010; Nano et al. 2017; Cosgrave et al. 2021). The combined effects of OSA and insomnia in COMISA patients further exacerbate the risk to cardiovascular health.

In this context, heart rate variability (HRV) biomarkers may provide clinically relevant information to support phenotyping in COMISA patients (Martín‐Montero et al. 2025).

Despite the known physiological impacts of insomnia and OSA individually, only a limited number of studies have examined HRV in patients with COMISA, and further research is still needed to better understand the autonomic alterations associated with this comorbidity and their clinical implications. Preliminary findings suggest that COMISA patients may exhibit compounded or distinct patterns of autonomic dysregulation, further complicating the clinical profile and treatment strategies (Martín‐Montero et al. 2025; Sweetman et al. 2021; Ma et al. 2024).

Recent studies have explored the existence of alternative frequency bands that more effectively characterize the impact of OSA on HRV (Martín‐Montero et al. 2023; Martín‐Montero, Gutiérrez‐Tobal, Gozal, et al. 2021; Martín‐Montero, Gutiérrez‐Tobal, Kheirandish‐Gozal, et al. 2021; Martín‐Montero et al. 2022). New evidence has also highlighted the effects of insomnia and COMISA on HRV using these same bands (Martín‐Montero et al. 2025), suggesting that, in COMISA and OSA patients, BWMS, BWCOMISA, and BWRes tend to decrease, whereas BWOSA tends to increase.

The objective of this study is to characterize and compare the physiological and sleep profiles associated with COMISA, insomnia, and OSA in population‐based datasets from Portugal and Spain. By leveraging polysomnographic (PSG) and HRV data, we aim to identify distinctive patterns across these conditions and explore potential inter‐country differences in sleep and autonomic regulation.

## 2 Methodology

### 2.1 Study Populations and Data Sources

Two datasets were used in this study: Hospital de São João, Porto, Portugal, and Hospital Universitario Río Hortega, Valladolid, Spain. Adults (≥ 18 years) with available data on apnea‐hypopnea index (AHI), insomnia status, body mass index (BMI), daytime sleepiness (assessed using the Epworth Sleepiness Scale (ESS)), sleep efficiency, nocturnal symptoms (snoring, witnessed apneas, and gasping), and in‐laboratory PSG I with ECG signals were included. PSG recordings from both hospitals were scored according to the American Academy of Sleep Medicine (AASM) criteria (version 3) (Troester et al. 2023), using standard definitions of apnea (≥ 90% airflow reduction for ≥ 10 s) and hypopnea (≥ 30% airflow reduction for ≥ 10 s associated with ≥ 3% oxygen desaturation or arousal). The AHI was calculated as the total number of apneas and hypopneas divided by total sleep time. Given that HRV was analysed, and to ensure focus only on COMISA effects, individuals with cardiovascular diseases were excluded.

### 2.2 Group Classification Criteria

Patients were classified into four categories, namely insomnia, OSA, COMISA and healthy. These groups were created according to:

- Insomnia was determined, in both hospitals, based on clinical diagnosis documented in the medical records or ongoing pharmacological treatment for sleep, as patients were not directly contacted for standardized assessment.
- OSA was diagnosed based on clinical guidelines (Epstein et al. 2009), using either (1) an AHI ≥ 5 events/h combined with symptoms; or (2) an AHI ≥ 15 events/h, regardless of symptoms.
- COMISA was identified in patients who met the criteria for both insomnia and OSA, using the same AHI thresholds and clinical diagnosis.

### 2.3 Signal Preprocessing and HRV Extraction

The HRV methodology followed the procedure described by Martín‐Montero, Gutiérrez‐Tobal, Kheirandish‐Gozal, et al. (2021), using frequency ranges adapted for adults as described in Martín‐Montero et al. (2025). ECG signals were first processed to detect R‐peaks, after which artefact correction was applied to obtain normal‐to‐normal (N‐N) intervals. Physiologically implausible beats were identified and removed according to the criteria proposed by Penzel et al. (2003): exclusion of R‐R intervals outside the range 0.33 s < R‐R < 1.5 s, and exclusion of R‐R intervals differing by more than 0.66 s from the preceding interval. These rejection thresholds ensured that only valid N‐N intervals were retained for HRV analysis. Time‐ and frequency‐domain HRV metrics were extracted and conventional time‐domain HRV metrics were computed to assess ANS function and short‐term fluctuations, with the following features:

- SDANN: Standard deviation (SD) of the average of N‐N intervals across 5‐min segments, reflecting long‐term HRV changes. Lower values may indicate reduced autonomic flexibility.
- SDNNI: Mean of the SDs of N‐N intervals within 5‐min segments, indicating short‐term HRV influenced by both sympathetic (SNS) and parasympathetic (PNS) activity.
- RMSSD: Root mean square of successive differences between N‐N intervals, a metric closely associated with vagal (PNS) tone.
- pNN50: Percentage of adjacent N‐N intervals differing by more than 50 ms, also reflecting parasympathetic function.
- mHR: Mean heart rate (in beats per minute), reflecting the combined influence of SNS (increasing HR) and PNS (reducing HR) activity.

Regarding the frequency‐domain, as N‐N intervals are not evenly spaced in time, the HRV time series was resampled to a uniform frequency of 4 Hz to enable spectral analysis. Power spectral density was estimated using Welch's periodogram method (Welch 1967), employing a Hamming window of 1200 samples (corresponding to 5‐min segments at 4 Hz), with 50% overlap and a fast Fourier transform of 2^11^ points. Five‐minute epochs were selected for frequency analysis, as this duration represents the maximum interval over which signal stationarity can reasonably be assumed for spectral HRV analysis (Penzel et al. 2003). Classical bands (very low frequency—VLF: 0–0.04 Hz; low frequency—LF: 0.04–0.15 Hz; high frequency—HF: 0.15–0.40 Hz; and LF/HF ratio and normalized LF—LFn) and specific bands (BWMS: 0.0005–0.0045 Hz, BWOSA: 0.011–0.049 Hz, BWCOMISA: 0.071–0.11 Hz and BWRes) were analysed, and the relative power within each band was computed.

This methodology enables detailed characterization of whole night ANS activity regardless of sleep stages in specific frequency domains, offering insight into physiological differences across populations, including those affected by insomnia, OSA and COMISA.

### 2.4 Statistical Analysis

To compare the datasets, the following variables were analysed: age, gender, BMI, ESS score, sleep efficiency, AHI, snoring, witnessed apneas and gasping. Continuous variables (age, BMI, ESS, sleep efficiency and AHI) were categorized into discrete groups to enable comparison across datasets. BMI was classified according to the standard World Health Organization (WHO) categories (WHO 2025), and AHI according to previously mentioned clinical guidelines (Epstein et al. 2009). Age was categorized into four groups: 18–24 years, 25–39 years, 40–59 years, ≥ 60 years, while ESS into two groups, based on established clinical interpretations (ESS, n.d.): < 10 normal daytime sleepiness and ≥ 11 excessive daytime sleepiness. Finally, sleep efficiency was categorized based on Ahn et al. (2024) in < 75%, ≥ 75%–< 85%, ≥ 85%.

Normality of continuous variables was assessed using the Shapiro–Wilk test. Variables with a normal distribution are presented as mean ± standard deviation (SD), while non‐normally distributed variables are presented as median and interquartile range (IQR). Categorical variables were compared using Fisher's Exact Test. For continuous variables, group comparisons were performed using ANOVA when data were normally distributed and the Kruskal–Wallis test when they were not. A *p*‐value < 0.05 was considered statistically significant. When significant differences were found, pairwise comparisons were performed, and *p*‐values were adjusted for multiple comparisons using the Benjamini–Hochberg false discovery rate (FDR) procedure. Effect sizes were calculated using epsilon‐squared ($\epsilon^{2}$) for Kruskal–Wallis tests, eta‐squared ($\eta^{2}$) for ANOVA and Cramer's *V* for Fisher's exact tests.

## 3 Results

After applying the inclusion and exclusion criteria, 147 patients remained in the Portuguese dataset and 264 in the Spanish dataset. Tables containing the results are provided in Appendices A, B, C.

### 3.1 Comparative Analysis of the Portuguese and Spanish Datasets

The characteristics and distributions of the Portuguese and Spanish datasets are summarized in Table A1 of Appendix A.

Gender distribution shows a marked and statistically significant difference (*p* ≤ 0.01), with a much higher proportion of males in the Spanish dataset (73%) compared to Portugal (40%).

In terms of age, both datasets were well‐matched (*p* = 0.255), with median ages in the low‐to‐mid 50s and similar age group distributions.

BMI distributions were similar across both countries (*p* = 0.157), with median values indicating overweight status in both groups. Nevertheless, Portugal had a higher proportion of individuals with Class I obesity (35% vs. 23%), whereas Spain had slightly higher rates of Class II and III obesity.

The Portuguese patients reported significantly higher levels of daytime sleepiness as indicated by the ESS score (*p* = 0.015), with 69% classified as having excessive daytime sleepiness versus 57% in Spain. This subjective sleep reduction is reinforced by lower sleep efficiency in the Portuguese group (82.9% vs. 88.8%, *p* ≤ 0.01). Moreover, a greater proportion of Portuguese patients had sleep efficiency below 75% (34% vs. 15%).

The most striking difference between the datasets is in disease severity, which was significantly higher in the Spanish dataset (*p* ≤ 0.01). The median AHI in Spain was 38.1 events/h (severe OSA), compared to 11.1 events/h (mild OSA) in Portugal. Over half (58%) of Spanish patients fell into the severe OSA category, while 35% of Portuguese patients had normal AHI values.

Snoring (97% vs. 89%; *p* = 0.002) and gasping (53% vs. 25%; *p* ≤ 0.01) were significantly more common in Portuguese patients, while witnessed apneas showed no between‐group difference (64% vs. 61%; *p* = 0.527).

### 3.2 Comparisons Across Groups

#### 3.2.1 Portuguese Dataset

The comparison of clinical variables, distribution of respiratory events and HRV measures across the four groups (healthy, insomnia, OSA and COMISA) are presented in Tables A2, A3, A4 of Appendix B, respectively. No significant differences were observed for gender distribution (*p* = 0.333), while age varied significantly across groups (*p* = 0.003, *V* = 0.240), with COMISA patients being the oldest (median: 59 years), followed by those with OSA (median: 52 years). Post hoc comparisons indicated that the COMISA group differed significantly from healthy and insomnia groups.

BMI also varied significantly (*p* ≤ 0.01, *V* = 0.295), with the COMISA group presenting a median value within the class I obesity range, while the healthy group showed a median value within the overweight range. Post hoc analysis indicated, once again, differences between the COMISA groups and healthy and insomnia patients.

Daytime sleepiness, measured with the ESS, was significantly different (*p* = 0.002, *V* = 0.363), with the highest sleepiness reported in the OSA (median: 14) and COMISA (median: 13) groups. Surprisingly, the insomnia group showed the lowest ESS score. Similar to age and BMI, post hoc analysis confirmed that the COMISA group differed significantly from both the healthy and insomnia groups.

Sleep efficiency was significantly lower in COMISA (*p* = 0.033, *V* = 0.221), with values dropping to a median of 77.1% compared to 86.7% in the healthy group. Meanwhile, the insomnia and OSA groups showed a comparable distribution of sleep efficiency. However, post hoc analysis revealed no statistically significant differences between the groups.

AHI, as expected, was significantly different (*p* ≤ 0.01, *V* = 0.586), with COMISA (median: 18.1 events/h) and OSA (12.9 events/h) groups having higher values than healthy and insomnia groups. Post hoc comparisons confirmed that both OSA and COMISA groups differed significantly from healthy and insomnia groups, while not differing significantly from each other.

Event counts mirrored this, with total events, apneas, and hypopneas higher in OSA and COMISA (all *p* ≤ 0.01, $\epsilon^{2}=0.486$, $\epsilon^{2}=0.149$ and $\epsilon^{2}=0.474$, respectively). Post hoc comparisons showed that OSA and COMISA differed significantly from the insomnia group in all event counts. Notably, both OSA and COMISA exhibited a hypopnea‐dominant profile, with COMISA showing an even stronger predominance of hypopneas compared to OSA. However, the healthy group exhibited a higher total number of events than the insomnia group, with a similar number of apneas but a greater number of hypopneas.

Symptom reports (snoring, witnessed apneas, gasping) were highly prevalent in all disturbed groups but did not differ significantly (*p* = 0.080, 0.773, 0.105, respectively).

Despite substantial differences in sleep quality and respiratory burden, HRV and autonomic indices did not significantly differ across groups. Neither time‐domain measures (SDANN, SDNNI, RMSSD, pNN50) nor frequency‐domain indices (VLF, LF, HF, LF/HF, LFn) showed significant differences across the groups. However, the HF band was lower in the OSA group, and the mHR was highest in the OSA group (68.9 bpm), followed closely by COMISA (68.1 bpm). The specific bands (BWOSA, BWCOMISA, BWMS, BWRes) also did not show significant differences across groups, but BWOSA exhibited the highest values in the OSA group.

#### 3.2.2 Spanish Dataset

The comparison of clinical variables, distribution of respiratory events, and HRV measures across the four groups is presented in Tables A5, A6, A7 of Appendix B, respectively.

Age differed significantly across groups (*p* = 0.019, *V* = 0.160), with the insomnia and COMISA patients being older on average (62 and 57 years, respectively) than those in the healthy (46 years) and OSA (54 years) groups. Post hoc comparisons indicated that the healthy group varied significantly from the OSA and COMISA groups.

BMI also showed significant group differences (*p* = 0.009, *V* = 0.195). The OSA group had the highest median BMI, within the overweight category, and a greater proportion of patients in the class II and III obesity categories, a pattern also observed in COMISA. The insomnia group had the second‐highest average BMI, but, unlike OSA and COMISA, did not include any patients in the class III obesity category. Post hoc comparisons showed that BMI differed significantly between the OSA and healthy groups.

Daytime sleepiness was significantly different across groups (*p* ≤ 0.01, *V* = 0.255), with the highest scores in the COMISA (72%) and OSA groups (59%). As in the Portuguese population, the insomnia group exhibited the lowest ESS scores. Post hoc comparisons indicated that the OSA and COMISA groups differed significantly from both the healthy and insomnia groups. Despite these differences, sleep efficiency did not vary significantly across groups.

As expected, AHI differed strongly between groups (*p* ≤ 0.01, *V* = 0.579), with OSA showing the highest median value (47.9 events/h), followed by COMISA (34.4), while healthy and insomnia patients remained within the normal to mild range (4.8 and 9.8, respectively). Post hoc comparisons confirmed that both OSA and COMISA groups differed significantly from healthy and insomnia groups, while not differing significantly from each other.

Event counts closely reflected these differences (all *p* ≤ 0.01). Post hoc comparisons indicating that OSA and COMISA groups differed significantly from the healthy and insomnia groups. Event composition further distinguished the groups, with OSA patients showing the highest number of events, followed by COMISA. Consistent with the Portuguese dataset, both OSA and COMISA patients exhibited a hypopnea‐dominant profile, which was more pronounced in the COMISA group (71.3%), despite its lower total event counts.

Nocturnal symptoms (snoring, witnessed apneas and gasping) did not differ significantly across groups.

Classical HRV measures in the time and frequency domains did not show statistically significant differences between groups, although certain trends were noted. The COMISA and insomnia groups exhibited higher mHR values, and in addition, the OSA group showed lower HF values. For specific bands, BWOSA differed significantly (*p* = 0.019, $\eta^{2}=0.037$). It was highest in OSA (mean = 0.366), intermediate in COMISA (=0.329), and lower in healthy (=0.313) and insomnia (=0.265), with post hoc comparisons indicating a significant difference between OSA and healthy groups. BWMS, BWCOMISA and BWRes showed no significant differences (*p* = 0.102, 0.088, 0.071, respectively), though BWRes showed a near‐trend towards lower values in OSA and COMISA.

### 3.3 Joint Populations

After analysing the Portuguese and Spanish datasets separately, we then joined them for a combined analysis. The comparison of clinical variables, distribution of respiratory events, and HRV measures across the four groups is presented in Tables A8, A9, A10 of Appendix B, respectively.

When the two populations were merged, the distribution of clinical variables largely reflected an intermediate profile between the Portuguese and Spanish datasets.

Age and BMI remained significant differentiating factors across groups (*p* = 0.001, *V* = 0.153 and *p* ≤ 0.01, *V* = 0.199, respectively), with COMISA patients being older and showing higher obesity prevalence, as in the Portuguese dataset. Post hoc comparison shows differences between OSA and healthy groups for both variables.

Daytime sleepiness and sleep efficiency differed significantly between groups (*p* ≤ 0.01, *V* = 0.277 and *p* = 0.002, *V* = 0.162, respectively) with COMISA patients having the highest ESS score, as in the Spanish dataset, and lowest sleep efficiency, as in the Portuguese dataset. Post hoc comparisons further highlighted differences between the groups, particularly in daytime sleepiness.

By definition, all healthy and insomnia patients had normal AHI values (median < 5 events/h). OSA patients showed the highest AHI (median 41.6 events/h), while COMISA patients displayed moderate severity (median 22.6 events/h). When comparing this findings with the separate analyses, OSA patients also showed the highest AHI values in the Spanish dataset, whereas in the Portuguese dataset the highest values were observed among COMISA patients. These differences were highly significant (*p* ≤ 0.01, *V* = 0.602). Post hoc confirmed distinct AHI levels across the four groups.

Event counts followed the same pattern as AHI (all *p* ≤ 0.01), with the OSA group showing the highest number of total events (median: 250), followed by the COMISA group (median: 123). As expected, the healthy and insomnia groups had the lowest event counts, although the healthy group showed more events than the insomnia group, which was also observed in the Portuguese dataset. Both OSA and COMISA groups exhibited a hypopnea‐dominant profile, more pronounced in COMISA, consistent with the separate dataset findings. Post hoc comparisons show differences between groups, especially for apneas, where all four groups differed significantly from each other.

When the Portuguese and Spanish datasets were combined, several classic HRV features in both the time and frequency domains showed significant differences, including RMSSD, mHR, VLF, HF, LF/HF and LFn. Among the features, mHR (*p* = 0.049, $\epsilon^{2}=0.012$) was highest in the COMISA group (69.1 bpm), followed by the OSA group (68.7 bpm). The HF band (*p* ≤ 0.01, $\epsilon^{2}=0.040$) showed the lowest values in the OSA group, consistent with the findings from the separate analyses. Both LF/HF and LFn (*p* ≤ 0.01, $\epsilon^{2}=0.037$) reached their highest values in the OSA group. Post hoc comparisons showed that mHR was the only variable with no statistically significant differences between groups.

All specific bands showed significant differences across groups. BWMS (*p* = 0.028, $\epsilon^{2}=0.015$) reached its lowest values in the OSA and COMISA groups. Consistent with the separate dataset analyses, BWOSA (*p* ≤ 0.01, $\epsilon^{2}=0.094$) was highest in the OSA group. BWCOMISA (*p* ≤ 0.01, $\epsilon^{2}=0.043$) showed the lowest values in the insomnia and COMISA groups. Finally, BWRes also exhibited small but significant differences among groups (*p* = 0.001, $\epsilon^{2}=0.031$). Post hoc comparisons further showed differences between groups for all specific bands, except BWMS, for which no statistically significant differences between groups were observed.

## 4 Discussion

This comparative analysis between Portuguese and Spanish datasets reveals important differences and similarities in demographic, clinical and sleep‐related variables, shedding light on possible cultural, diagnostic, or systemic differences influencing sleep disorder presentations across the two populations.

The age distribution across populations suggests comparability in terms of life stage and age‐related risk.

The greater proportion of males in the Spanish dataset, together with the higher risk of OSA in men (Senaratna et al. 2017; Young et al. 1993; Lozo et al. 2017), may partly explain the greater AHI severity observed in this group. In contrast, the Portuguese population presented with milder disease but reported more frequent subjective symptoms, such as snoring and gasping, and reported higher daytime sleepiness and lower sleep efficiency, indicating greater sleep fragmentation. Minor variations in BMI median and obesity classes distribution may also have contributed subtly to differences in OSA severity (Young et al. 1993; Unal et al. 2019) and sleep efficiency (Shochat et al. 2016; Koolhaas et al. 2019; Carneiro‐Barrera et al. 2020).

The higher ESS scores and lower sleep efficiency in the Portuguese population may suggest greater functional impairment (D'Rozario et al. 2020) despite lower AHI levels, pointing towards a potential mismatch between objective severity and subjective symptom burden (Gurkan et al. 2024; Mediano et al. 2007).

When comparing Tables A2 and A5, both datasets confirm age and obesity as key distinguishing factors across the defined groups (Unal et al. 2019; Romero‐Corral et al. 2010; Ghavami et al. 2023; Hirani and Smiley 2023; Wu et al. 2024). The post hoc comparisons further refine this interpretation. In the Portuguese dataset, the significant differences in age and BMI were mainly driven by the COMISA group, reinforcing the view of COMISA as a clinically more burdened phenotype. In the Spanish dataset, these differences were more strongly explained by contrasts between healthy and OSA patients, suggesting that age and BMI may be more closely linked to respiratory severity in that population. Importantly, the associated effect sizes were small to moderate, indicating that these factors contribute meaningfully to group differentiation, although they are not the only determinants of the observed clinical profiles.

Although obesity is prevalent in the insomnia group within our datasets, supporting a possible association, current research indicates that the evidence linking obesity and insomnia remains insufficient (Chan et al. 2018).

Daytime sleepiness is higher in both OSA and COMISA, as seen in literature (Zhang et al. 2021; Subramanian et al. 2021), across the two datasets, though overall levels are consistently higher in the Portuguese sample. This aligns with the Portuguese dataset's lower sleep efficiency values, which may help explain the higher ESS scores observed. The difference between daytime sleepiness and sleep efficiency suggests that subjective complaints and arousal‐related disturbances, represented by the ESS score in insomnia, OSA and COMISA, may not be fully reflected by global efficiency metrics of sleep efficiency (Gurkan et al. 2024; Mediano et al. 2007). The post hoc results also help clarify that daytime sleepiness is not uniformly distributed across all groups. In both individual datasets, the main contrasts were driven by OSA and COMISA relative to healthy and insomnia groups. The effect sizes for ESS category comparisons were moderate in both datasets, suggesting that this variable has a relevant role in distinguishing clinical phenotypes, particularly those with greater respiratory burden.

In both datasets, the insomnia groups consistently show the lowest ESS scores, suggesting that subjective reports of daytime sleepiness may not fully capture the extent of impairment in insomnia. Interestingly, in the Spanish dataset, the insomnia group also demonstrates the lowest sleep efficiency, which appears contradictory to their low ESS scores. While reduced sleep efficiency reflects fragmented or insufficient nocturnal rest, the low ESS values indicate that these patients do not perceive themselves as excessively sleepy during the day (Manber et al. 2021; Fasiello et al. 2024).

AHI differs markedly between the two populations, with higher values and a greater proportion of patients classified as severe in the Spanish dataset, as mentioned before. The overall higher AHI values, and consequently greater OSA severity, in the Spanish population may be attributed to differences in diagnostic procedures. At Hospital Universitario Río Hortega, in‐lab PSG is the first line exam, whereas at Hospital de São João, it is PSG level III. This is due to the high waiting list in Portugal, where in‐lab PSG is reserved for patients unable to complete PSG III at home or those with inconclusive PSG III results. This methodological difference likely contributes to the lower AHI values observed in the Portuguese population, given that most of the results are obtained from at‐home exams, which are not contemplated within these datasets.

Event counts, which reached statistical significance in both datasets, closely reflect the respiratory burden of OSA and COMISA. As expected, event counts are higher in the Spanish dataset, consistent with its overall higher severity profile. In the Portuguese population, the healthy group showing a greater number of respiratory events compared to the insomnia group suggests that even individuals classified as ‘healthy’ may experience a higher frequency of respiratory events that do not reach the diagnostic threshold for OSA (Roeder et al. 2020). By contrast, the insomnia group exhibited fewer total events, reinforcing that their sleep difficulties are primarily related to initiation and maintenance rather than respiratory disturbances. Moreover, since insomnia patients typically experience shorter total sleep durations, this may also contribute to the lower number of detected respiratory events. The statistical significance of these variables is expected, as they are directly associated with AHI, which also showed significance in both datasets.

The higher hypopnea‐dominant profile in COMISA is observed in both datasets and has been previously reported in literature (Kim et al. 2023). One possible physiological explanation is increased nocturnal arousals and a lower arousal threshold in patients with COMISA. In a recent study, Brooker et al. (2023) reported that individuals with COMISA exhibit a higher nocturnal arousal index and lower values of a parameter they identified as the arousal threshold than patients with OSA alone. A lower value of this parameter may predispose patients to more frequent sleep fragmentation and to the occurrence of respiratory events that meet hypopnea criteria, including associated arousals. This mechanism may contribute to the hypopnea‐dominant profile observed in COMISA in our study.

The post hoc findings are consistent with the expected role of respiratory burden as the main discriminator between groups. In both Portuguese and Spanish datasets, healthy and insomnia groups clustered together, while OSA and COMISA clustered together for AHI, confirming that the presence of sleep‐disordered breathing, rather than insomnia symptoms alone, drives these differences. As expected, given that group classification was based on AHI thresholds, the large effect sizes observed for AHI category comparisons reflect the underlying diagnostic criteria, reinforcing the central role of respiratory burden in separating the four clinical groups.

The absence of statistically significant differences in classical HRV measures across the time and frequency domains prevents us from establishing clear evidence of ANS imbalance or dysfunction in the populations when analysed separately. Nevertheless, the higher mHR observed in the Portuguese OSA group, followed closely by COMISA, and in the Spanish insomnia and COMISA groups suggests an increased cardiovascular load associated with these conditions (Martín‐Montero et al. 2025; Dissanayake et al. 2021; Cosgrave et al. 2021; Ma et al. 2024). Moreover, in both datasets, OSA groups show lower HF values, agreeing with previous work (Martín‐Montero et al. 2025; Ha and Kim 2022; Aydin et al. 2004).

While no specific HRV bands reached significance in the Portuguese dataset, BWOSA emerged as a discriminative biomarker in the Spanish dataset, aligning with respiratory event load and supporting its potential as a spectral marker of OSA, further reinforced by the post hoc statistical difference observed between the OSA and healthy groups. Although the overall effect size for BWOSA in the Spanish dataset was small, this result still suggests a contribution of this band to group differentiation, particularly in relation to the respiratory burden of OSA. A similar trend was observed in the Portuguese dataset, where, although statistical significance was not reached, higher BWOSA values in OSA remain consistent with previous literature and reflect the sympathetic activation triggered by apneic events. The significant difference in BWOSA observed exclusively in the Spanish population may be due to the higher OSA severity within this group. This interpretation is consistent with previous studies showing that BWOSA activity increases with the severity of apnea (Martín‐Montero et al. 2025; Martín‐Montero, Gutiérrez‐Tobal, Kheirandish‐Gozal, et al. 2021). In contrast, in both datasets the remaining bands showed no significant differences, diverging from prior reports that had suggested their potential as discriminative markers (Martín‐Montero et al. 2025, 2023; Martín‐Montero, Gutiérrez‐Tobal, Gozal, et al. 2021; Martín‐Montero, Gutiérrez‐Tobal, Kheirandish‐Gozal, et al. 2021; Martín‐Montero et al. 2022).

When combining both datasets, age and obesity consistently emerge as significant risk factors, reinforcing their established role in disease susceptibility. Daytime sleepiness and sleep efficiency demonstrate patterns comparable to those observed in the individual populations, while AHI values also align with the same overall profile. Notably, given that the Spanish dataset shows a higher incidence of severe OSA, the joint analysis yields intermediate values between the Portuguese and Spanish populations for both the OSA and COMISA groups. As expected, the analysis also highlights the predominance of hypopnea‐driven events, in line with the previously observed event‐count distribution. The persistence of post hoc differences involving healthy and OSA groups supports the robustness of age and BMI as markers, while AHI post hoc comparisons further showed the clear separation between the healthy/insomnia groups and the OSA and COMISA groups. In the combined dataset, the effect sizes for age and BMI remained small, whereas AHI showed a large effect size, which is consistent with its role in defining the diagnostic groups.

The main difference between the individual analyses and the combined dataset lies in the HRV features. While some trends are similar to those observed in the separate analyses, several associations now reach statistical significance. Notably, OSA and COMISA patients demonstrate a higher cardiovascular burden, as indicated by elevated mHR, and OSA patients show greater sympathetic activity, reflected in the higher BWOSA. Measures such as LF/HF ratio and LFn, which provide insights into sympathovagal balance, also differ significantly between groups. The higher values observed in OSA patients, consistent with previous findings, reflect a sympathovagal imbalance with increased sympathetic predominance, which is a characteristic of autonomic dysfunction in OSA (Gula et al. 2003; Aydin et al. 2004; Sequeira et al. 2019). These statistically significant differences highlight ANS imbalance and dysfunction in the combined dataset.

The post hoc comparisons further nuance these findings by showing that not all significant HRV variables translated into clear pairwise group separation. For example, mHR reached statistical significance in the combined analysis, but no post hoc differences were identified, indicating that its global effect was weak and likely distributed across groups. Similarly, BWMS was significant overall, yet without significant pairwise contrasts, suggesting limited usefulness as a stand‐alone differentiator. In contrast, BWOSA, BWCOMISA, HF, LF/HF and LFn showed clearer post hoc patterns, supporting their stronger potential to capture clinically meaningful autonomic differences between phenotypes. Among all these significant HRV changes, BWOSA was associated with the greatest effect size. This suggests that BWOSA may be the most robust HRV‐derived discriminator among the significant markers in the combined analysis.

Additionally, BWMS, which shows its lowest values in the OSA and COMISA groups, follows patterns previously reported in the literature (Martín‐Montero et al. 2025). As BWMS is associated with the structure of macro‐arousals in sleep, its decreased activity in these groups appears to be a primary consequence of OSA, reflecting alterations in both sleep architecture and autonomic balance. Consistent with earlier observations, BWOSA demonstrates higher values in the OSA group, a finding also evident in the separate analyses. Regarding BWCOMISA, it reached statistically significant differences, with the reduced activity in the COMISA group confirming the impaired PNS modulation during sleep in this population reported by Martín‐Montero et al. (2025), highlighting this feature as a potential specific biomarker of the COMISA population. Overall, in this combined dataset, all specific bands demonstrate potential as biomarkers to differentiate between clinical groups.

From a clinical perspective, BWOSA and BWCOMISA may have potential clinical utility as non‐invasive biomarkers derived from ECG signals to support screening, phenotyping and monitoring of sleep disorders. Specifically, BWOSA could complement conventional indices such as AHI in assessing disease severity or treatment response, while BWCOMISA may help identify patients with persistent autonomic dysregulation characteristic of COMISA, enabling more personalized management.

These findings should be interpreted within the broader context of interactions between co‐occurring sleep disorders, as recent evidence suggests that certain sleep‐related conditions may coexist without clear causal links. For example, a recent meta‐analysis reported no significant association between OSA and sleep bruxism, despite their frequent co‐occurrence (Blaszczyk, Waliszewska‐Prosół, et al. 2024). Similarly, PSG evidence suggests that sleep bruxism does not occur more frequently in COMISA compared to OSA or control populations, and that conventional PSG‐derived metrics alone may not be sufficient to distinguish between these phenotypes (Blaszczyk, Cruz, et al. 2024). In line with this, emerging clinical perspectives describe COMISA as a distinct phenotype characterized by a compounded pathophysiological burden, in which insomnia‐related hyperarousal and OSA‐related intermittent hypoxia interact to increase overall symptom severity and clinical complexity (Cruz et al. 2025). Taken together, these findings reinforce the notion that COMISA should be interpreted as a complex clinical entity arising from overlapping but not necessarily directly coupled mechanisms, rather than as a simple additive condition, while also suggesting that PSG‐derived metrics alone may be insufficient for accurate phenotyping, thereby supporting the clinical relevance of complementary biomarkers, such as HRV‐derived features.

The joint analysis of the dataset reveals results consistent with the separate analyses for most clinical variables. However, it demonstrates significant differences in HRV features, both in the time and frequency domains. By merging the Portuguese and Spanish datasets, the analysis produces values that appear to balance the characteristics of each population, resulting in intermediate patterns. This effect is particularly evident in variables such as OSA severity and autonomic function, where the higher incidence of severe OSA in the Spanish dataset influences the overall distribution, while the Portuguese dataset contributes a comparatively milder profile. Consequently, the combined analysis provides a more robust and representative picture of the clinical and autonomic alterations associated with OSA and COMISA across populations, while also highlighting the sensitivity of HRV markers to cross‐population variability.

This study presents some limitations. Notably, the Spanish insomnia group consisted of only 7 patients, none of whom reported excessive daytime sleepiness, which may partially explain the lower ESS scores observed in this group. Additionally, differences in the PSG acquisition and scoring procedures across datasets may have influenced the results, as may demographic, clinical, or cultural differences in the study population. Furthermore, sleep stage differentiation was not considered in the present analysis, which could provide additional insights into HRV dynamics. Therefore, incorporating sleep stage‐specific analysis represents an important direction for future research. Finally, multiple statistical comparisons across several HRV metrics may increase the risk of Type I errors. Although a post hoc FDR correction was applied to mitigate this issue, the results should still be interpreted with caution and warrant confirmation in larger cohorts. In addition, the physiological mechanisms underlying the hypopnea‐dominant profile observed in COMISA remain incompletely understood and should be further investigated in future studies.

## 5 Conclusion

The results confirmed age and obesity as distinguishing factors across the defined groups. Although AHI values differed significantly between datasets, with the Spanish dataset presenting a much higher median AHI, both confirmed a more hypopnea‐dominant profile in COMISA.

In terms of HRV, neither separate dataset showed significant group differences in standard time‐ or frequency‐domain indices, although trends were consistent with decreased parasympathetic activity in OSA and elevated heart rate in COMISA. This convergence suggests that classical HRV metrics are not robust discriminators across these populations.

Interestingly, while no specific HRV bands reached significance in the Portuguese dataset, BWOSA emerged as a discriminative biomarker in the Spanish dataset, aligning with respiratory event load and supporting its potential as a spectral marker of OSA.

In the joint Portuguese and Spanish analysis, several HRV measures showed significant group differences, revealing autonomic imbalance. COMISA patients had the highest heart rate, as well as markedly low BWCOMISA activity, reflecting ANS impairment. All specific bands also reached statistically significant differences, confirming their potential as spectral HRV biomarkers in the context of both OSA and COMISA.

Together, these findings highlight the complementary burdens of insomnia and OSA in COMISA, while also demonstrating that population and methodological differences can influence which markers best differentiate between groups.

## Author Contributions

**Margarida Carvalho:** writing – original draft, data curation, formal analysis, methodology, visualization, software. **Eva Almeida:** data curation, writing – review and editing, validation. **Pedro Amorim:** data curation, writing – review and editing, validation. **Marta Drummond:** data curation, investigation, validation. **Adrián Martín‐Montero:** data curation, software, validation, methodology, supervision, writing – review and editing. **Gonzalo C. Gutiérrez‐Tobal:** data curation, software, validation, methodology, writing – review and editing, project administration, funding acquisition. **Tomás Ruiz‐Albi:** data curation, investigation, validation. **Julio De Frutos:** data curation, investigation, validation. **Pedro Pereira Rodrigues:** conceptualization, resources, funding acquisition, project administration. **Daniela Ferreira‐Santos:** conceptualization, investigation, formal analysis, supervision, writing – review and editing, funding acquisition, project administration.

## Funding

This work was supported by Interreg, 0043_NET4SLEEP_2_E.

## Conflicts of Interest

The authors declare no conflicts of interest.

## Data Availability

The data that support the findings of this study are available on request from the corresponding author. The data are not publicly available due to privacy or ethical restrictions.

## Appendix A

### Comparative Analysis of the Portuguese and Spanish Datasets

**TABLE A1:** Comparison of clinical variables between patients of the Portuguese and Spanish datasets.

Variable	Portugal	Spain	*p*
Number	147	264	
Gender (Male)	58 (40)	194 (73)	≤ **0.01**
Age			
Median (IQR)/Mean ± SD	53 (45–63)	54 ± 13	0.255
18–24	6 (4)	4 (2)	
25–39	15 (10)	36 (14)	
40–59	77 (53)	128 (48)	
≥ 60	49 (33)	96 (36)	
BMI (kg/m^2^)			
Median (IQR)	29.7 (26.2–32.9)	29.4 (25.8–33.2)	0.157
Underweight	1 (1)	1 (1)	
Healthy weight	27 (18)	53 (20)	
Overweight	47 (32)	100 (38)	
Class I obesity	52 (35)	62 (23)	
Class II obesity	11 (8)	30 (11)	
Class III obesity	9 (6)	18 (7)	
ESS score			
Median (IQR)	13 (9–15)	11 (9–12)	**0.015**
Normal daytime sleepiness^1^	45 (31)	114 (43)	
Excessive daytime sleepiness^2^	102 (69)	150 (57)	
Sleep efficiency (%)			
Median (IQR)	82.9 (70.4–89.1)	88.8 (80.4–94.8)	≤ **0.01**
< 75%	50 (34)	40 (15)	
75%–85%	33 (22)	60 (23)	
≥ 85%	64 (44)	164 (62)	
AHI (events/h)			
Median (IQR)	11.1 (5.7–20.6)	38.1 (17.9–64.8)	≤ **0.01**
Healthy	51 (35)	40 (15)	
Mild	42 (29)	12 (5)	
Moderate	39 (26)	58 (22)	
Severe	15 (10)	154 (58)	
Snoring	143 (97)	235 (89)	**0.002**
Witnessed apneas	94 (64)	160 (61)	0.527
Gasping	78 (53)	66 (25)	≤ **0.01**

*Note:* Values are presented as median (IQR) or mean ± SD when indicated, and as number of patients with percentage in parentheses: *N* (%). The *p*‐value for group comparisons is reported, with bold values indicating statistical significance.

^1^ ESS score < 10.

^2^ ESS score ≥ 11.

## Appendix B

### Comparisons Across Groups

- Portuguese Dataset

**TABLE A2:** Comparison of clinical variables among patients in the four diagnostic groups (Healthy, Insomnia, OSA, COMISA) in the Portuguese dataset.

Variable	Healthy	Insomnia	OSA	COMISA	*p*
Number	17	34	31	65	
Gender (Male)	8 (47)	12 (35)	16 (52)	22 (34)	0.333
Age					
Median (IQR)	47 (43–52)^a^	49 (43–56)^a^	52 (41–58)^a,b^	59 (50–67)^b^	**0.003**, *V* = 0.240
18–24	3 (18)	2 (6)	1 (3)	0	
25–39	1 (6)	2 (6)	5 (16)	7 (11)	
40–59	10 (58)	23 (68)	18 (58)	26 (40)	
≥ 60	3 (18)	7 (20)	7 (23)	32 (49)	
BMI (kg/m^2^)					
Median (IQR)	26.2 (22.9–29.7)^a^	28.8 (25.2–30.5)^a^	28.6 (25.4–32.5)^a,b^	31.6 (29.0–33.8)^b^	≤ **0.01**, *V* = 0.295
Underweight	0	1 (3)	0	0	
Healthy weight	8 (47)	7 (21)	6 (19)	6 (9)	
Overweight	5 (29)	15 (44)	11 (36)	16 (25)	
Class I obesity	1 (6)	10 (29)	9 (29)	32 (49)	
Class II obesity	0	0	2 (6)	9 (14)	
Class III obesity	3 (18)	1 (3)	3 (10)	2 (3)	
ESS score					
Median (IQR)	10 (7–13)^a^	9 (4–13)^a^	14 (11–18)^a,b^	13 (12–15)^b^	**0.002**, *V* = 0.363
Normal daytime sleepiness^1^	9 (53)	18 (53)	8 (26)	10 (15)	
Excessive daytime sleepiness^2^	8 (47)	16 (47)	23 (74)	55 (85)	
Sleep efficiency (%)					
Median (IQR)	86.7 (80.9–93.1)^a^	84.2 (75.5–89.4)^a^	83.9 (76.6–89.6)^a^	77.1 (66.5–86.8)^a^	**0.033**, *V* = 0.221
< 75%	3 (18)	8 (24)	8 (26)	31 (48)	
75%–85%	2 (12)	10 (29)	10 (32)	11 (17)	
≥ 85%	12 (70)	16 (47)	13 (42)	23 (35)	
AHI (events/h)					
Median (IQR)	4.9 (2.7–9.3)^a^	3.6 (2.6–6.0)^a^	12.9 (10.2–24.2)^b^	18.1 (10.9–25.3)^b^	≤ **0.01**, *V* = 0.586
Healthy	17 (100)	34 (100)	0	0	
Mild	0	0	17 (55)	25 (39)	
Moderate	0	0	9 (29)	30 (46)	
Severe	0	0	5 (16)	10 (15)	
Snoring	16 (94)	33 (97)	29 (94)	65 (100)	0.080
Witnessed apneas	9 (53)	22 (65)	20 (65)	43 (66)	0.773
Gasping	7 (41)	24 (71)	14 (45)	33 (51)	0.105

*Note:* Values are presented as median (IQR), and as number of patients with percentage in parentheses: *N* (%). The *p*‐value for group comparisons is reported, with bold values indicating statistical significance. For statistically significant results, the effect size is reported after the *p*‐value. Superscript letters (a, b) above columns indicate the results of post hoc multiple comparisons corrected using the FDR. Groups sharing the same letter are not significantly different, whereas groups with different letters are significantly different (*p* < 0.05, FDR‐adjusted).

^1^ ESS score < 10.

^2^ ESS score ≥ 11.

**TABLE A3:** Distribution of respiratory events in the Portuguese dataset across the four groups (Healthy, Insomnia, OSA and COMISA).

Variable	Healthy	Insomnia	OSA	COMISA	*p*
Total number of events (apneas + hypopneas)	34 (15–57)^a^	21 (16–30)^a^	73 (57–140)^b^	93 (60–153)^b^	≤ **0.01**, $\epsilon^{2}=0.486$
Total number of apneas	1 (0–10)^a,c^	1 (0–2)^a^	14 (2–35)^b^	7 (1–25)^b,c^	≤ **0.01**, $\epsilon^{2}=0.149$
Total number of hypopneas	33 (9–48)^a^	20 (13–30)^a^	57 (49–94)^b^	81 (48–108)^b^	≤ **0.01**, $\epsilon^{2}=0.474$
% of apnea	2.9	4.8	19.2	7.5	
% of hypopnea	97.1	92.9	78.1	87.1	

*Note:* The number of events is presented as median (IQR). The relative contribution of apneas and hypopneas is expressed as percentages. The *p*‐value for group comparisons is reported, with bold values indicating statistical significance. For statistically significant results, the effect size is reported after the *p*‐value. Superscript letters (a, b, c) above columns indicate the results of post hoc multiple comparisons corrected using the FDR. Groups sharing the same letter are not significantly different, whereas groups with different letters are significantly different (*p* < 0.05, FDR‐adjusted).

**TABLE A4:** HRV measures in the Portuguese dataset across the four groups (Healthy, Insomnia, OSA and COMISA).

Variable	Healthy	Insomnia	OSA	COMISA	*p*
SDANN	0.064 (0.041–0.080)	0.059 (0.045–0.075)	0.058 (0.043–0.074)	0.063 (0.048–0.085)	0.901
SDNNI	0.075 (0.064–0.097)	0.070 (0.057–0.095)	0.067 (0.056–0.085)	0.079 (0.058–0.103)	0.719
RMSSD	0.076 (0.059–0.104)	0.067 (0.053–0.099)	0.065 (0.053–0.102)	0.079 (0.054–0.113)	0.519
pNN50	0.078 (0.031–0.141)	0.066 (0.033–0.115)	0.055 (0.028–0.110)	0.071 (0.036–0.163)	0.727
mHR	66.8 (61.5–70.1)	63.3 (60.8–73.7)	68.9 (61.8–75.7)	68.1 (60.8–74.1)	0.616
VLF	0.453 ± 0.146	0.453 ± 0.133	0.473 ± 0.145	0.418 ± 0.118	0.236
LF	0.241 ± 0.039	0.222 ± 0.046	0.217 ± 0.058	0.234 ± 0.046	0.234
HF	0.228 ± 0.104	0.248 ± 0.095	0.221 ± 0.091	0.250 ± 0.077	0.425
LF/HF	1.020 (0.812–1.665)	0.942 (0.673–1.201)	0.972 (0.740–1.352)	0.916 (0.717–1.205)	0.454
LFn	0.505 (0.448–0.625)	0.485 (0.402–0.545)	0.493 (0.425–0.575)	0.478 (0.418–0.546)	0.454
BWMS	0.090 (0.067–0.110)	0.082 (0.068–0.123)	0.095 (0.069–0.110)	0.075 (0.061–0.095)	0.189
BWOSA	0.258 ± 0.079	0.254 ± 0.068	0.273 ± 0.086	0.258 ± 0.075	0.749
BWCOMISA	0.051 (0.047–0.062)	0.051 (0.041–0.060)	0.046 (0.037–0.056)	0.048 (0.040–0.059)	0.424
BWRes	0.003 (0.002–0.003)	0.003 (0.003–0.005)	0.003 (0.002–0.003)	0.003 (0.002–0.004)	0.213

*Note:* Values are presented as median (IQR) or mean ± SD, conforming to the normality test results. The *p*‐value for group comparisons is reported, with bold values indicating statistical significance.

B Spanish Dataset

**TABLE A5:** Comparison of clinical variables among patients in the four diagnostic groups (Healthy, Insomnia, OSA, COMISA) in the Spanish dataset.

Variable	Healthy	Insomnia	OSA	COMISA	*p*
Number	33	7	188	36	
Gender (Male)	21 (64)	5 (71)	145 (77)	23 (64)	0.170
Age					
Mean ± SD	46 ± 15^a^	62 ± 9^a,b^	54 ± 13^b^	57 ± 11^b^	0.019, *V* = 0.160
18–24	1 (3)	0	2 (1)	1 (3)	
25–39	11 (33)	0	23 (12)	2 (6)	
40–59	13 (40)	2 (29)	97 (52)	16 (44)	
≥ 60	8 (24)	5 (71)	66 (35)	17 (47)	
BMI (kg/m^2^)					
Median (IQR)	25.3 (24.0–29.4)^a^	28.9 (23.8–31.2)^a,b^	29.7 (26.7–34.4)^b^	28.5 (25.9–30.8)^a,b^	0.009, *V* = 0.195
Underweight	1 (3)	0	0	0	
Healthy weight	13 (39)	3 (43)	29 (15)	8 (22)	
Overweight	12 (37)	1 (14)	71 (38)	16 (44)	
Class I obesity	7 (21)	2 (29)	44 (24)	9 (25)	
Class II obesity	0	1 (14)	27 (14)	2 (6)	
Class III obesity	0	0	17 (9)	1 (3)	
ESS score					
Median (IQR)	9 (7–11)^a,b^	8 (8–9)^a^	11 (10–13)^b,c^	12 (10–13)^c^	≤ 0.01, *V* = 0.255
Normal daytime sleepiness^1^	20 (61)	7 (100)	77 (41)	10 (28)	
Excessive daytime sleepiness^2^	13 (39)	0	111 (59)	26 (72)	
Sleep efficiency (%)					
Median (IQR)	86.4 (76.0–94.8)	84.5 (69.5–90.4)	90.0 (81.9–95.2)	87.8 (77.7–93.4)	0.399
< 75%	7 (21)	3 (43)	24 (13)	6 (17)	
75%–85%	8 (24)	1 (14)	44 (23)	7 (19)	
≥ 85%	18 (55)	3 (43)	120 (64)	23 (64)	
AHI (events/h)					
Median (IQR)	4.8 (2.7–11.0)^a^	9.8 (5.9–12.9)^a^	47.9 (26.7–74.4)^b^	34.4 (24.3–60.0)^b^	≤ 0.01, *V* = 0.579
Healthy	33 (100)	7 (100)	0	0	
Mild	0	0	9 (5)	3 (8)	
Moderate	0	0	47 (25)	11 (31)	
Severe	0	0	132 (70)	22 (61)	
Snoring	25 (76)	6 (86)	172 (91)	32 (89)	0.062
Witnessed apneas	14 (42)	4 (57)	118 (63)	24 (67)	0.136
Gasping	6 (18)	0	51 (27)	9 (25)	0.373

*Note:* Values are presented as median (IQR) or mean ± SD conforming to the normality test results, and as number of patients with percentage in parentheses: *N* (%). The *p*‐value for group comparisons is reported, with bold values indicating statistical significance. For statistically significant results, the effect size is reported after the *p*‐value. Superscript letters (a, b, c) above columns indicate the results of post hoc multiple comparisons corrected using the FDR. Groups sharing the same letter are not significantly different, whereas groups with different letters are significantly different (*p* < 0.05, FDR‐adjusted).

^1^ ESS score < 10.

^2^ ESS score ≥ 11.

**TABLE A6:** Distribution of respiratory events in the Spanish dataset across the four groups (Healthy, Insomnia, OSA and COMISA).

Variable	Healthy	Insomnia	OSA	COMISA	*p*
Total number of events (apneas + hypopneas)	27 (17–62)^a^	44 (37–66)^a^	294 (165–476)^b^	209 (147–346)^b^	≤ **0.01**, $\epsilon^{2}=0.353$
Total number of apneas	6 (1–11)^a^	1 (0–4)^a^	74 (16–179)^b^	52 (23–105)^b^	≤ **0.01**, $\epsilon^{2}=0.239$
Total number of hypopneas	21 (14–50)^a^	44 (33–62)^a^	160 (97–275)^b^	149 (102–214)^b^	≤ **0.01**, $\epsilon^{2}=0.293$
% of apneas	22.2	2.3	25.0	24.9	
% of hypopneas	77.8	100.0	54.3	71.3	

*Note:* The number of events is presented as median (IQR). The relative contribution of apneas and hypopneas is expressed as percentages. The *p*‐value for group comparisons is reported, with bold values indicating statistical significance. For statistically significant results, the effect size is reported after the *p*‐value. Superscript letters (a, b) above columns indicate the results of post hoc multiple comparisons corrected using the FDR. Groups sharing the same letter are not significantly different, whereas groups with different letters are significantly different (*p* < 0.05, FDR‐adjusted).

**TABLE A7:** HRV measures in the Spanish dataset across the four groups (Healthy, Insomnia, OSA and COMISA).

Variable	Healthy	Insomnia	OSA	COMISA	*p*
SDANN	0.061 (0.045–0.075)	0.058 (0.057–0.077)	0.061 (0.048–0.082)	0.056 (0.044–0.076)	0.422
SDNNI	0.070 (0.055–0.080)	0.064 (0.050–0.128)	0.073 (0.053–0.104)	0.078 (0.058–0.107)	0.481
RMSSD	0.055 (0.043–0.071)	0.076 (0.039–0.160)	0.057 (0.039–0.105)	0.073 (0.043–0.119)	0.589
pNN50	0.082 (0.028–0.123)	0.034 (0.019–0.127)	0.075 (0.024–0.152)	0.082 (0.026–0.146)	0.946
mHR	64.5 (59.0–70.9)	70.5 (66.1–73.2)	68.7 (62.9–74.1)	70.2 (61.7–76.8)	0.104
VLF	0.528 (0.427–0.616)	0.308 (0.272–0.510)	0.518 (0.381–0.614)	0.453 (0.302–0.626)	0.226
LF	0.248 (0.204–0.273)	0.267 (0.249–0.292)	0.241 (0.199–0.283)	0.248 (0.215–0.277)	0.650
HF	0.200 (0.128–0.258)	0.311 (0.155–0.340)	0.169 (0.105–0.249)	0.192 (0.104–0.292)	0.556
LF/HF	1.206 (0.928–1.955)	0.840 (0.749–2.227)	1.419 (0.886–2.241)	1.454 (0.834–2.130)	0.725
LFn	0.564 ± 0.125	0.541 ± 0.181	0.583 ± 0.146	0.574 ± 0.160	0.807
BWMS	0.085 (0.070–0.112)	0.064 (0.054–0.089)	0.072 (0.048–0.099)	0.069 (0.038–0.095)	0.102
BWOSA	0.313 ± 0.089^a^	0.265 ± 0.106^a,b^	0.366 ± 0.130^b^	0.329 ± 0.132^a,b^	**0.019**, *η* ^2^ = 0.037
BWCOMISA	0.083 (0.069–0.107)	0.078 (0.075–0.087)	0.073 (0.052–0.087)	0.075 (0.060–0.093)	0.088
BWRes	0.003 (0.002–0.004)	0.003 (0.002–0.004)	0.002 (0.001–0.004)	0.002 (0.002–0.003)	0.071

*Note:* Values are presented as median (IQR) or mean ± SD, conforming to the normality test results. The *p*‐value for group comparisons is reported, with bold values indicating statistical significance. For statistically significant results, the effect size is reported after the *p*‐value. Superscript letters (a, b) above columns indicate the results of post hoc multiple comparisons corrected using the FDR. Groups sharing the same letter are not significantly different, whereas groups with different letters are significantly different (*p* < 0.05, FDR‐adjusted).

## Appendix C

### Joint Populations

**TABLE A8:** Comparison of clinical variables among patients in the four diagnostic groups (Healthy, Insomnia, OSA, COMISA) in the Portuguese and Spanish datasets together.

Variable	Healthy	Insomnia	OSA	COMISA	*p*
Number	50	41	219	101	
Gender (Male)	29 (58)^a,b^	17 (41)^a^	161 (76)^b^	45 (45)^a^	≤ **0.01**, *V* = 0.282
Age					
Median (IQR)	47 (34; 54)^a^	51 (45; 61)^a,b^	55 (46; 62)^b^	59 (51; 66)^b^	**0.001**, *V* = 0.153
18–24	4 (8)	2 (5)	3 (1)	1 (1)	
25–39	12 (24)	2 (5)	28 (13)	9 (9)	
40–59	23 (46)	25 (61)	115 (53)	42 (41)	
≥ 60	11 (22)	12 (29)	73 (33)	49 (49)	
BMI (kg/m^2^)					
Median (IQR)	25.4 (23.5; 29.6)^a^	28.9 (24.7; 31.1)^a,b^	29.7 (26.6; 34.2)^b^	30.3 (27.2; 33.0)^b^	
Underweight	1 (2)	1 (2)	0	0	≤ **0.01**, *V* = 0.199
Healthy weight	21 (42)	10 (25)	35 (16)	14 (14)	
Overweight	17 (34)	16 (39)	82 (38)	32 (32)	
Class I obesity	8 (16)	12 (30)	53 (24)	41 (40)	
Class II obesity	0	1 (2)	29 (13)	11 (11)	
Class III obesity	3 (6)	1 (2)	20 (9)	3 (3)	
ESS score					
Median (IQR)	10 (7; 12)^a^	9 (5; 13)^a^	11 (10; 13)^b^	13 (11; 15)^c^	≤ **0.01**, *V* = 0.277
Normal daytime sleepiness^1^	29 (58)	25 (61)	85 (39)	20 (20)	
Excessive daytime sleepiness^2^	21 (42)	16 (39)	134 (61)	81 (80)	
Sleep efficiency (%)					
Median (IQR)	86.7 (77.5; 94.2)^a,b^	84.2 (73.5; 89.6)^a,b^	88.8 (81.1; 94.4)^a^	81.6 (70.0; 90.5)^b^	**0.002**, *V* = 0.162
< 75%	10 (20)	11 (27)	32 (14)	37 (37)	
75%–85%	10 (20)	11 (27)	54 (25)	18 (18)	
≥ 85%	30 (60)	19 (46)	133 (61)	46 (45)	
AHI (events/h)					
Median (IQR)	4.9 (2.8; 10.2)^a^	3.8 (2.7; 6.7)^a^	41.6 (21.7; 67.6)^b^	22.6 (14.2; 35.6)^c^	≤ **0.01**, *V* = 0.602
Healthy	50 (100)	41 (100)	0	0	
Mild	0	0	26 (12)	28 (28)	
Moderate	0	0	56 (25)	41 (40)	
Severe	0	0	137 (63)	32 (32)	
Snoring	41 (82)^a^	39 (95)^a^	201 (92)^a^	97 (96)^a^	**0.035**, *V* = 0.153
Witnessed apneas	23 (46)	26 (63)	138 (63)	67 (66)	0.102
Gasping	13 (26)^a^	24 (59)^b^	65 (30)^a^	42 (42)^a,b^	**0.001**, *V* = 0.200

*Note:* Values are presented as median (IQR), and as number of patients with percentage in parentheses: *N* (%). The *p*‐value for group comparisons is reported, with bold values indicating statistical significance. For statistically significant results, the effect size is reported after the *p*‐value. Superscript letters (a, b, c) above columns indicate the results of post hoc multiple comparisons corrected using the FDR. Groups sharing the same letter are not significantly different, whereas groups with different letters are significantly different (*p* < 0.05, FDR‐adjusted).

^1^ ESS score < 10.

^2^ ESS score ≥ 11.

**TABLE A9:** Distribution of respiratory events in the Portuguese and Spanish datasets together across the four groups (Healthy, Insomnia, OSA and COMISA).

Variable	Healthy	Insomnia	OSA	COMISA	*p*
Total number of events (apneas + hypopneas)	31 (17; 62)^a^	23 (16; 41)^a^	250 (137; 436)^b^	123 (81; 198)^c^	≤ **0.01**, $\epsilon^{2}=0.486$
Total number of apneas	4 (1; 11)^a^	1 (0; 2)^b^	57 (14; 155)^c^	19 (3; 53)^d^	≤ **0.01**, $\epsilon^{2}=0.316$
Total number of hypopneas	25 (12; 50)^a^	22 (15; 37)^a^	145 (82; 245)^b^	95 (62; 149)^c^	≤ **0.01**, $\epsilon^{2}=0.410$
% of apnea	12.9	4.3	22.8	15.4	
% of hypopnea	80.6	95.7	58.0	77.2	

*Note:* The number of events is presented as median (IQR). The relative contribution of apneas and hypopneas is expressed as percentages. The *p*‐value for group comparisons is reported, with bold values indicating statistical significance. For statistically significant results, the effect size is reported after the *p*‐value. Superscript letters (a, b, c, d) above columns indicate the results of post hoc multiple comparisons corrected using the FDR. Groups sharing the same letter are not significantly different, whereas groups with different letters are significantly different (*p* < 0.05, FDR‐adjusted).

**TABLE A10:** HRV measures in the Portuguese and Spanish datasets together across the four groups (Healthy, Insomnia, OSA and COMISA).

Variable	Healthy	Insomnia	OSA	COMISA	*p*
SDANN	0.061 (0.045; 0.076)	0.058 (0.049; 0.076)	0.060 (0.047; 0.082)	0.060 (0.046; 0.081)	0.799
SDNNI	0.072 (0.056; 0.084)	0.067 (0.055; 0.096)	0.072 (0.053; 0.103)	0.079 (0.058; 0.105)	0.576
RMSSD	0.059 (0.044; 0.086)^a,b^	0.066 (0.051; 0.100)^a,b^	0.059 (0.040; 0.107)^a^	0.079 (0.052; 0.116)^b^	**0.025**, $\epsilon^{2}=0.016$
pNN50	0.080 (0.031; 0.132)	0.061 (0.032; 0.118)	0.068 (0.025; 0.151)	0.076 (0.031; 0.151)	0.950
mHR	66.2 (59.0; 70.4)^a^	66.0 (60.9; 73.8)^a^	68.7 (62.9; 74.7)^a^	69.1 (60.8; 75.5)^a^	**0.049**, $\epsilon^{2}=0.012$
VLF	0.501 (0.366; 0.615)^a,b^	0.425 (0.342; 0.570)^a,b^	0.507 (0.381; 0.606)^a^	0.433 (0.327; 0.535)^b^	**0.002**, $\epsilon^{2}=0.030$
LF	0.240 (0.211; 0.272)	0.228 (0.191; 0.262)	0.236 (0.199; 0.281)	0.237 (0.209; 0.270)	0.793
HF	0.197 (0.133; 0.275)^a,b^	0.258 (0.187; 0.313)^a^	0.177 (0.109; 0.255)^b^	0.223 (0.174; 0.313)^a^	≤ **0.01**, $\epsilon^{2}=0.040$
LF/HF	1.197 (0.927; 1.822)^a,e^	0.919 (0.668; 1.250)^b^	1.337 (0.866; 2.176)^a,c^	0.993 (0.742; 1.551)^b,d,e^	≤ **0.01**, $\epsilon^{2}=0.037$
LFn	0.545 (0.481; 0.646)^a,e^	0.479 (0.401; 0.556)^b^	0.572 (0.464; 0.685)^a,c^	0.498 (0.426; 0.608)^b,d,e^	≤ **0.01**, $\epsilon^{2}=0.037$
BWMS	0.086 (0.068; 0.112)^a^	0.079 (0.064; 0.119)^a^	0.074 (0.051; 0.101)^a^	0.074 (0.055; 0.095)^a^	**0.028**, $\epsilon^{2}=0.015$
BWOSA	0.301 (0.229; 0.359)^a^	0.245 (0.196; 0.322)^b^	0.352 (0.261; 0.434)^c^	0.277 (0.222; 0.350)^a,b^	≤ **0.01**, $\epsilon^{2}=0.094$
BWCOMISA	0.072 (0.051; 0.092)^a^	0.054 (0.043; 0.067)^b^	0.068 (0.048; 0.085)^a^	0.055 (0.043; 0.073)^b^	≤ **0.01**, $\epsilon^{2}=0.043$
BWRes	0.003 (0.002; 0.004)^a^	0.003 (0.002; 0.005)^a^	0.003 (0.002; 0.004)^b^	0.003 (0.002; 0.004)^a,b^	**0.001**, $\epsilon^{2}=0.031$

*Note:* Values are presented as median (IQR) or mean ± SD, conforming to the normality test results. The *p*‐value for group comparisons is reported, with bold values indicating statistical significance. For statistically significant results, the effect size is reported after the *p*‐value. Superscript letters (a, b, c, d, e) above columns indicate the results of post hoc multiple comparisons corrected using the FDR. Groups sharing the same letter are not significantly different, whereas groups with different letters are significantly different (*p* < 0.05, FDR‐adjusted).
